# Unexplained bacterial meat spoilage during the moose hunting in northern Norway – a review of cases 2008–2021

**DOI:** 10.1186/s13028-023-00683-0

**Published:** 2023-07-03

**Authors:** Terje Domaas Josefsen, Torill Mørk, Anders Aarthun Ims

**Affiliations:** 1 Faculty of Bioscience and Aquaculture, Nord University, NO-8026 Bodø, Norway; 2 Section of Food Safety and Animal Health Research, Norwegian Veterinary Institute, NO-9016 Tromsø, Norway; 3 Finnmark Estate, NO-9513 Alta, Norway

**Keywords:** *Alces*, Clostridia, Histology, Questionnaire, Shooting, Slaughter

## Abstract

**Background:**

Sudden and unexpected spoilage of moose (*Alces alces*) carcasses has incidentally been reported in northern Norway. Hunters describe a strong foul odour and greenish discolouration of moose carcasses, hence the nickname “green moose”. Finnmark Estate has registered all reported cases of “green moose” in Finnmark county in the period 2008–2021. In 2013, a questionnaire was introduced to gather more detailed information. Bacteriological and histological examinations were performed on submitted samples of spoiled moose meat. The aim of the present report is to summarize the data gathered about the “green moose” cases, and to discuss possible causes.

**Results:**

Ninety-three valid cases of “green moose” meat spoilage were registered in Finnmark county, giving this form of meat spoilage a prevalence of 0.85% of hunted moose. The carcass weights of spoiled carcasses were within normal weights for moose carcasses in Finnmark. Adult bulls were significantly more, and calves were less frequently affected by meat spoilage. No distinct geographical pattern or “hotspots” could be identified, but multiple cases in the same hunting area same year were reported. The meat spoilage was detected within 5 h after shooting in five cases, and 53% of cases were detected within 2 days after shooting. The meat spoilage was primarily found in deep muscle groups. Bacteriological analyses of 13 samples of spoiled meat were not conclusive. A mixture of aerobic bacteria was detected in 12 samples, and swarming clostridia in 10 samples. Histological examination of seven samples showed abundance of bacteria in fasciae and connective tissue surrounding blood vessels. Injury shootings were not more frequent in “green moose” cases than in moose hunting in general. Other possibly predisposing events to meat spoilage were evisceration later than 60 min after shooting, delayed skinning and contamination by ruminal content. Whether these events occurred more often in “green moose” than normal moose was difficult to determine, due to lack of reference data.

**Conclusions:**

Based on the bacteriological results and the characteristics of the meat spoilage we suggest that clostridia are a main factor involved. How and why clostridia are spread to the muscles and causing the often rapid meat spoilage, is unexplained.

**Supplementary Information:**

The online version contains supplementary material available at 10.1186/s13028-023-00683-0.

## Background

Hunting is in principle the killing and processing of a wild animal under field conditions, without slaughterhouse facilities and equipment. For moose (*Alces alces*), the normal procedure is to shoot the moose by use of a rifle with expanding bullet. A minimum calibre of 6.5 mm and a minimum impact energy of 2.2 kJ are required [1]. The moose should be shot through the chest, injuring lungs, heart and/or major blood vessels, resulting in loss of consciousness and death due to blood loss and circulatory collapse [2]. The moose may run for a short distance, in average 65 m for adult moose, before falling [3]. The slaughter is then started by immediate evisceration of the gastrointestinal tract with the carcass laying more or less at the place where it has fallen. Evisceration should be finished not later than one hour after shooting [4]. The carcass must then be transported to a place where skinning can be done, as this requires the possibility of hanging up the carcass by the hocks. Transport possibilities and distance in northern Norway vary considerably. The skinning facility may be a nearby shed or barn, or a field camp with carcasses hung up in self-made tripods [4] or other improvised hanging arrangements, protected against rain by a tarpaulin. After skinning, the carcass is usually allowed to hang for tenderization (ageing). In Norway the rule of thumb for length of the tenderization period is 40 day degrees (days multiplied by average temperature, e.g. 10 days at 4 °C). The carcass may be hung up whole for tenderization, or it may be cut up into major parts that are hung up separately.

Meat hygiene during moose hunting may be challenging [4, 5]. The shot may not hit as intended, resulting in injured animals, sometimes with punctured stomachs or intestines, staying alive for minutes or hours before dying or being killed by a second shot. Field conditions may make hygienic evisceration of a heavy animal difficult. Skinning may have to be delayed to protect the carcass during transport, thereby compromising rapid chilling. Tenderization is often subjected to ambient temperatures. Sauvala et al. [6] detected significantly more enterobacteria and *Escherichia coli* on moose than white-tailed deer carcasses. They suggest that this may be due to the more laborious handling of the much heavier moose carcasses.

The Norwegian Veterinary Institute (NVI), Tromsø regional laboratory, has since 2004 irregularly been contacted during the moose hunting season about cases of sudden and unexpected spoilage of moose meat. The hunters described greenish discolouration of fasciae and muscles and a strong foul odour arising from the moose carcass only few days after shooting. Cases were reported from all parts of the area served by the laboratory (Finnmark, Troms and northern parts of Nordland counties).

Analysis of submitted meat samples from these early cases (2004–2007) showed growth of common gastrointestinal bacteria like *E. coli*, other Enterobacteriaceae, enterococci and clostridia, and at NVI we at first suspected the meat spoilage to be the result of poor slaughter hygiene. However, the hunters claimed that they had done the slaughtering as they had always done, and that they never had experienced this kind of rapid meat spoilage before.

Finnmark county is the northernmost county of Norway, and many cases of meat spoilage occurred in this county. The moose hunting in Finnmark is entirely administered by Finnmark Estate (FeFo). The area is about 46 000 km^2^ (95% of the area of Finnmark county), and 700–800 moose are shot during the autumn hunting each year [7]. Hunters that had experienced this particular form of meat spoilage (soon named “green moose” due to the prominent greenish discolouration), started arguing with the hunting management at FeFo. They had paid a hunting fee to shoot the moose, and when the whole carcass had to be condemned, they wanted license to shoot another moose. FeFo then decided that hunters that could properly document this form of meat spoilage, would have the moose accepted as “fallen game” and get license to shoot another moose. Since 2008, FeFo have registered moose hunter’s reports of “green moose”. From 2013, a questionnaire was used to get more exact information about the cases. This report includes all data collected by FeFo, and also bacteriological and histological examinations of samples submitted to NVI, Tromsø, from different parts of northern Norway.

The purpose of this report is to summarize the data collected about “green moose” in the years 2008–2021, and to discuss possible causes of this particular form of meat spoilage.

## Methods

### Registration in Finnmark county

In the period 2008 to 2012, the following registrations were done when a case of “green moose” meat spoilage appeared: Date and place (municipality and hunting area) for shooting the moose, name of hunting team leader, exact time when the moose was shot, sex and age class (calf 0.5 year, young 1.5 years, and adult 2.5 years or more), carcass weight, condemnation (whole carcass or only parts), and type of documentation. Registration of date, exact time for shooting, carcass weight and calf sex are sometimes lacking in these early registrations (for exact data, see Additional file 1).

From 2013, a questionnaire was used to obtain more details about “green moose” cases. The questionnaire contained the same elements as the previous registration, but the following elements were added: Description of bullet hit point, time from shooting to evisceration, any contamination by gastrointestinal content, time from shooting to skinning, any abnormalities observed during evisceration and skinning, ambient temperature at the time of shooting and during the tenderization period, whether the carcass was hanged up whole for tenderization or divided into separate parts, exact time when they detected that the meat was spoiled, and which parts of the carcass that were spoiled.

The questionnaire was sent to hunters by email when they contacted FeFo about a case of “green moose”, and a completed questionnaire was usually received within a few days. Not all hunters returned a completed questionnaire, and by mistakes some hunters did not receive the questionnaire, thus explaining missing data in 9 cases (see Additional file 1).

The official hunting statistics for Finnmark county [7] was used to compare the spoiled and normal carcasses with respect to distribution of age and sex, distribution within municipalities and carcass weight.

### Bacteriology and histology

Sporadic submission of meat samples from cases of “green moose” meat spoilage occurred in the report period, reaching a total of 13 samples. The samples were submitted to NVI in Tromsø by the hunters themselves or by veterinarians or personnel from the Norwegian Food Safety Authority. The samples originated from the three northernmost counties in Norway (Finnmark, Troms and Nordland; Table 4). Handling of the samples prior to laboratory examination varied (Table 4), and was outside the control of the laboratory.

**Table 1:** Age and sex distribution of cases of moose meat spoilage (“green moose”) in Finnmark county, Norway, 2008–2021 compared to the distribution of age class and sex in all moose shot in Finnmark county in the same years^a^

	Calves (male or female) n (%)	Young bulls n (%)	Young cows n (%)	Adult bulls n (%)	Adult cows n (%)	Total n (%)
Meat spoilage (“green moose”)	7 (7.5%)	10 (10.8%)	12 (12.9%)	51 (54.8%)	13 (14.0%)	93 (100%)
Hunting statistics for Finnmark county 2008–2021	2 979 (27.6%)	1 731 (16.0%)	1 546 (14.3%)	2 993 (27.7%)	1 553 (14.4%)	10 802 (100%)

^a^ Hunting statistics from Hjorteviltregisteret [7]

The meat samples were examined for bacterial growth using the NVI standard operational procedure (SOP) for detection of pathogenic bacteria: after sterilisation of the sample surface by a gas flame, a small cut was made with a sterile scalpel, and a sterile plastic inoculation needle was used to transfer a minor amount of material from the sample to agar plates. Each sample was spread on blood agar and bromothymol blue lactose agar for aerobic incubation (37 °C) and blood agar for anaerobic incubation (37 °C). Anaerobic condition was created in an anaerobic jar, using AnaeroGen™ 2.5 L sachet (Thermo Fisher Scientific, Waltham, USA). Blood agar for aerobic incubation at room temperature (20 °C, not part of SOP) was included for samples 1–6 and 8 (Table 4). The incubation time was 18–24 h before first reading, and incubation continued until 48 h before termination. Bacteria were identified to genus or species, based on growth characteristics on agar plates, Gram staining, simple biochemical tests (catalase, oxidase, indole) and the use of test kits API 20 E, API 20 NE and rapid id 32 A v3.2 (BioMérieux, France).

Seven of the 13 meat samples (Table 4, samples 1–6 and 8) were prepared for histological examination. Representative specimens, 2 × 2 × 0.5 cm, were fixed in 10% phosphate buffered formalin, processed, embedded in paraffin, sectioned and routinely stained with haematoxylin-eosin, selected sections also Gram stained.

### Statistical analysis

Chi square test was used to compare the distribution of age class and sex of spoiled carcasses versus normal carcasses. Chi square test was also used to investigate whether the way of hanging up the carcass (whole or divided into parts) influenced the time to detection of meat spoilage (before or after two days) or the fate of the carcass (total or partial condemnation). A two-sided t-test was used to compare number of days until detection of meat spoilage at different ambient temperatures. All tests were carried out in Microsoft 365 Excel. Level of significance was P < 0.05.

## Results

### Registrated data

A total of 94 cases of “green moose” meat spoilage were registered by FeFo from 1.1.2008 to 31.12.2021. One case was excluded, as the moose was injured by a shot through the gastrointestinal tract and found dead the day after with signs of peritonitis. This report is based on the 93 remaining cases.

Cases of “green moose” occurred every year in the registration period, average 6.6 cases/year, range 3 to 12. In the same period a total of 10 802 moose were shot in Finnmark without being spoiled [7], making the overall prevalence of “green moose” 0.85%.

The 93 cases of “green mose” were distributed on age class and sex as shown in Table 1. Compared with the corresponding distribution of age and sex in the hunting statistics [7], adult bulls were overrepresented and calves were underrepresented among “green moose”. The difference in distribution between “green moose” and “normal moose” was statistically significant (P < 0.0001).

**Table 2:** Geographical variation in occurrence of moose meat spoilage (“green moose”) in Finnmark county, Norway, 2008–2021

Municipality/-ities	Cases of meat spoilage (“green moose”)	Number of moose shot	Prevalence of “green moose”
Alta	8	515	1.53
Berlevåg, Båtsfjord, Gamvik, Hammerfest, Nordkapp^a^	3	241	1.23
Porsanger	14	1268	1.09
Deanu/Tana	27	2829	0.95
Kárásjoga/Karasjok	25	2671	0.93
Sør-Varanger	6	748	0.80
Unjargga/Nesseby	4	794	0.50
Vadsø	1	250	0.40
Guovdageainnu/Kautokeino	4	1093	0.36
Lebesby	1	393	0.25

^a^ Five municipalities are grouped together, as very few moose are shot in these municipalities

Among the 93 “green moose” cases, 75 had registered carcass weight, either weighed (n = 59) or estimated (n = 16). The “green moose” and normal moose had similar carcass weights, the mean carcass weights of the hunting statistics [7] being within one SD of the mean “green moose” carcass weights (data in Additional file 1).

Of the 93 cases of “green moose” 56 (60.2%) was shot in September and 37 (39.8%) in October. Internal data at FeFo of 10 773 moose shootings in Finnmark county 2008–2021 showed similar distribution (58.8% in September, 40.5% in October and 0.7% in November).

The “green moose” meat spoilage resulted in the whole carcass being condemned in 71 cases, parts of the carcass in 19 cases and in three cases the final result of condemnation is unknown. In 12 cases of partial condemnation, the weight of the condemned parts was registered, ranging from 15 to 95 kg, mean 47.5 kg.

Cases of “green moose” were documented by the municipality wildlife administration (27), the Norwegian Food Safety Authority or authorized veterinarians (29), personnel at the official weight registration (24), by photos (3), or by sending samples for laboratory analysis (6). Four registered cases were without sufficient documentation, and those cases were not accepted as fallen game, but were still registered as “green moose”.

In general, most cases of meat spoilage occurred in the municipalities where most moose were shot. There was variation in prevalence between municipalities (Table 2), but it was difficult to see any distinct pattern in this variation. Guovdageainnu/Kautokeino municipality had particularly low prevalence while neighbouring municipalities Kárásjoga/Karasjok and Alta both had prevalence above mean.

**Table 3:** Parts of the moose carcass reported as “green moose” in questionnaire reports 2013–2021 (n = 45)

Affected parts	Number of cases	Total condemnation	Partial condemnation
Whole carcass (both fore and hind parts)	12	12	0
Fore parts (mainly neck and shoulder, may include back and parts of the trunk)	17	11	6
Hind parts (mainly steak, may include back and parts of the trunk)	12	7	5
Left side only (left shoulder and steak, left side of trunk)	1		1
Back only	1		1
Not specified	2^a^	1	
Sum	45	31	13

^a^ In one case the fate of carcass is unknown

Each municipality is divided into a variable number of hunting areas, the total number of hunting areas in Finnmark county being about 280. The 93 cases of meat spoilage occurred in 78 hunting areas. Most hunting areas (65) had only one case, 11 hunting areas had two cases and 2 hunting areas had three cases. Among the 11 hunting areas with two cases, both cases occurred the same year in 4 areas. Among the 2 hunting areas with three cases, all cases occurred the same year in one area, while two of three occurred the same year in the other area.

The occurrence of multiple cases in the same area in the same season is also reported to NVI Tromsø from an incident on the island Grytøya in Troms county where four cases of “green moose” meat spoilage occurred in 2020 (three samples submitted for bacteriology, Table 4). All four cases were detected 1–3 days after shooting, and all carcasses were subjected to total condemnation. The hunters report that at least back to 2003 no such incidence had occurred on the island, and no cases occurred in 2021.

### Questionnaire data

A total of 45 cases of “green moose” were reported by filling out the questionnaire or giving corresponding information in text.

Bullet hit point was in the preferred target area (thorax, included shoulder-area, lungs and heart) in 38 cases. In six cases, other hit points were reported: the thoracal vertebrae (3), the neck (2) and the head (1). One report did not specify hit point.

Time from shooting to evisceration was reported as 30 min or less in 34 cases (75.6%) and between 30 and 60 min in 8 cases (17.8%). In the remaining 3 cases, time to evisceration was reported to be 90, 105 and 120 min. The 120 min case was explained as caused by a suspected injury shooting, and the hunters waited for a dog to trace the moose. It appeared that the moose was hit by a shot through the lungs and found dead 230 m from where it was shot.

Contamination by ruminal content occurred in two cases, caused by the bullet perforating the cranial part of the rumen. The hunters claim that the spread of ruminal content was limited, and that they were able to clean the carcass properly. In the remaining 43 cases, no perforation of the gastrointestinal tract was reported during shooting or slaughtering.

Time from shooting to skinning was reported in 44 cases, and varied from 1 to 44 h. The reports divided into two main groups: Those that skinned the animal soon after shooting (15 reports, mean 3.1 h after shooting, range 1–8 h) and those that delayed skinning until next day (28 reports, mean 20.2 h, range 15–30 h). In one case, skinning was reported to take place 44 h after shooting.

Five hunting teams reported incidental findings during evisceration and skinning. The findings were low carcass weight, empty stomach, old rib fractures, parasite nodules in fasciae around the hocks and a discoloured area on the lower back of the carcass, considered to be a few days old bruise. The remaining 40 hunting teams reported no specific findings.

**Fig. 1:**
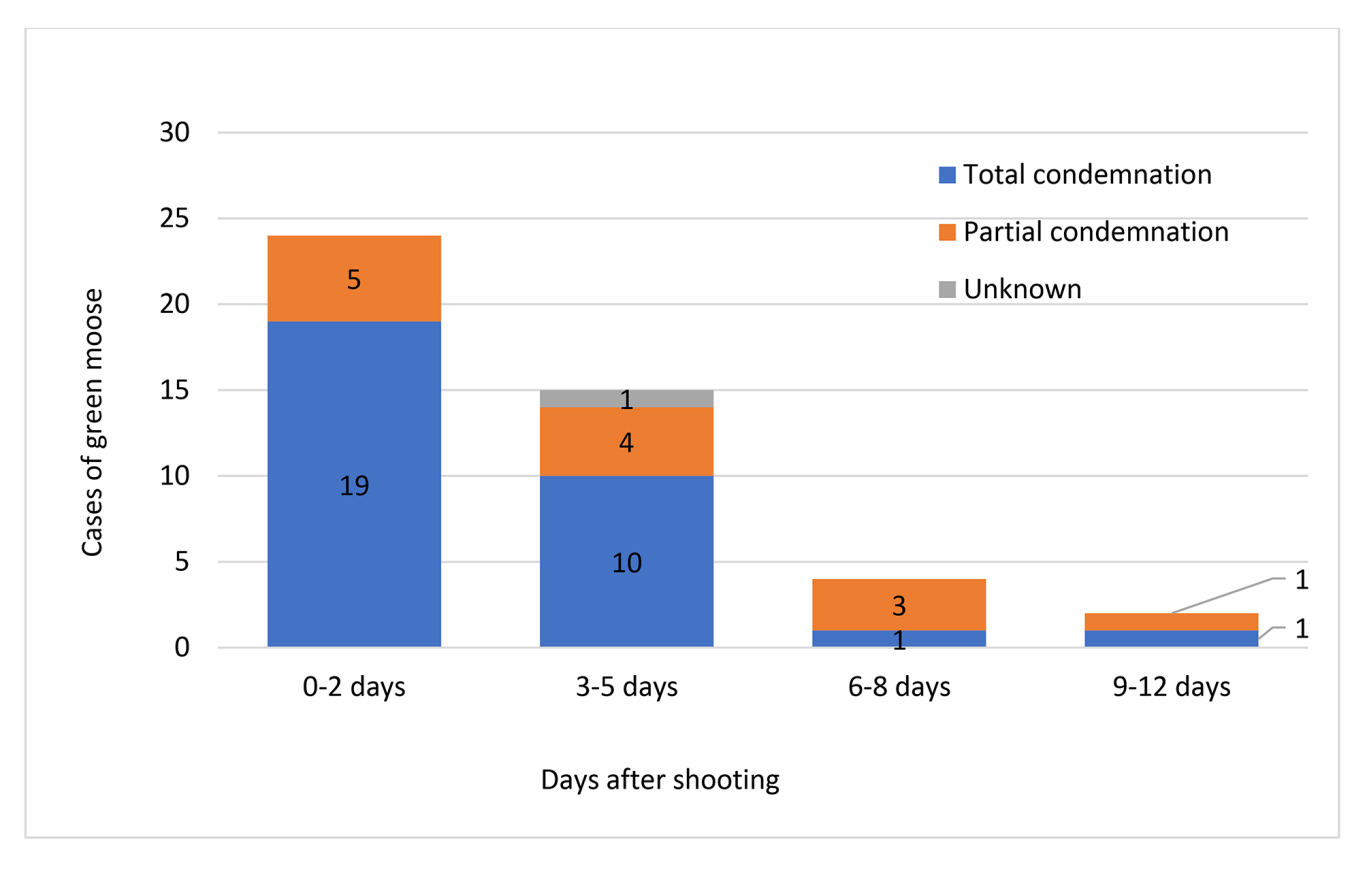
Number of days from shooting the moose to detection of meat spoilage, based on questionnaires filled in by the hunters 2013–2021 (n = 45). The fate of the carcass (total or partial condemnation) is indicated in each bar

Time from shooting to detection of meat spoilage is shown in Fig. 1. The earliest detection of “green moose” meat spoilage was foul odour during evisceration 10–15 min after shooting. The hunting team immediately proceeded to skinning and cutting up the carcass in order to possibly save some parts, but the whole carcass became spoiled. Four more cases were detected as early as 2–5 h after shooting. In one of these cases, foul odour and greenish discolouration were detected during skinning about two hours after shooting. In another case the hunters did not detect anything unnormal during evisceration and skinning, but foul odour and greenish discolouration were detected when they started to divide the carcass into parts between 2 and 3 h after shooting. In the last two cases foul odour was noticed during skinning 4–5 h after shooting. The hunters hung up the carcass for tenderization anyway. After 2–3 days the foul odour had become stronger and the greenish discolouration more evident.

Ten cases were detected during skinning 1 day after shooting, while nine more cases were detected 2 days after shooting. These cases were detected during hunter’s own inspection of the carcasses, during dividing the carcass into parts, and, in one case, during skinning two days after shooting.

Altogether 53% (24/45) of ”green moose” cases were detected within 2 days after shooting. The remaining cases were detected 3–12 days after shooting. The cases detected within 2 days more often resulted in condemnation of the whole carcass than cases detected later (Fig. 1).

**Fig. 2:**
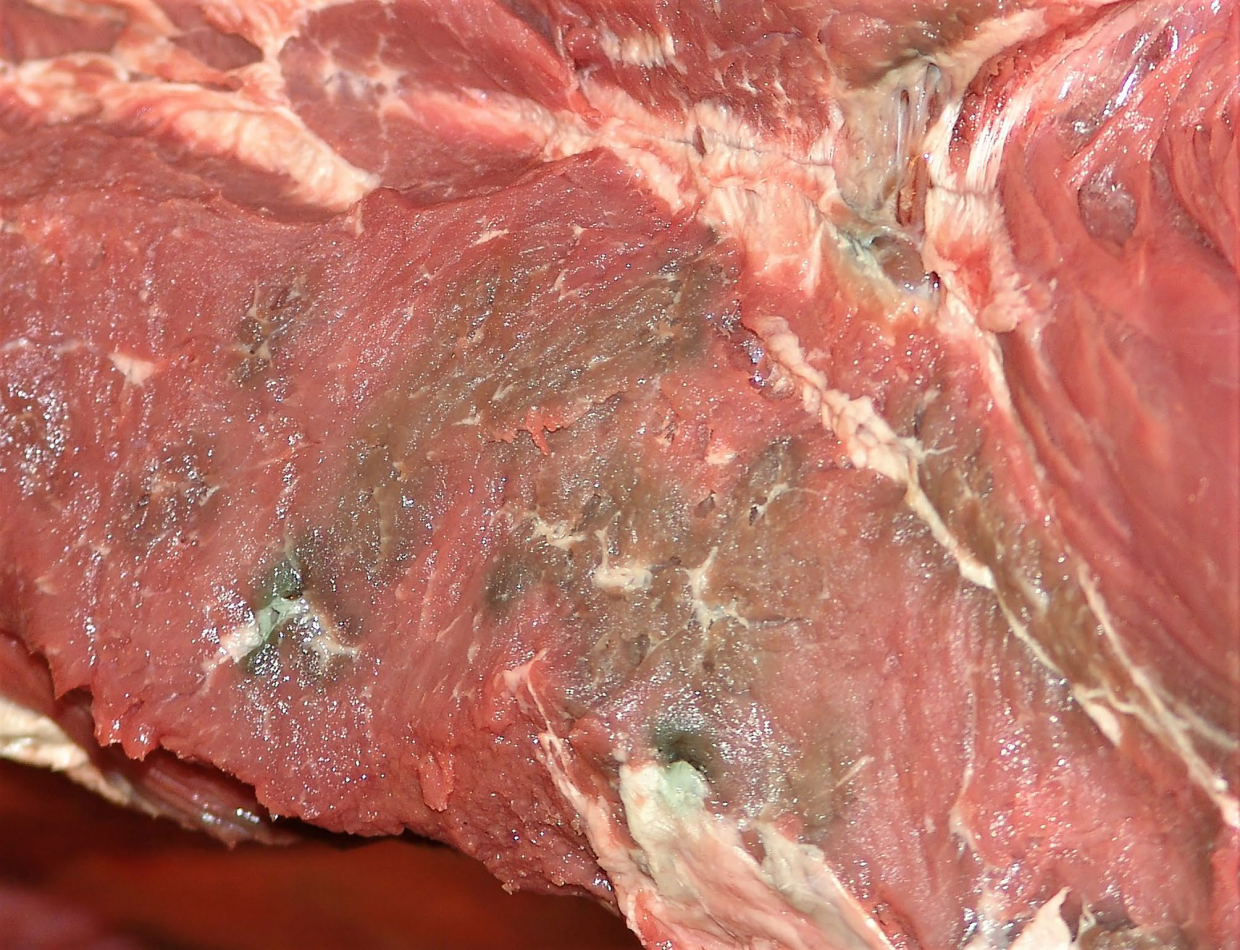
Incision through thigh muscle in a case of meat spoilage in an adult male moose (Sample No. 4 in Table 4). A mottled greenish discolouration is visible, apparently following blood vessels and intermuscular connective tissue

The hunters reported which parts were affected when they first discovered the meat spoilage. The answers are summarized in Table 3. The green discolouration was often described to be found in the depth of muscles, close to the cervical vertebrae of the neck or to the femoral bone of the thigh. Typically, the greenish discolouration was seen in muscle fasciae and fat. When extending into muscles the greenish discolouration appeared to follow blood vessels and intermuscular connective tissue, giving the cut surface of affected muscle a mottled appearance (Fig. 2). Five reports mentioned in particular a greenish discolouration of the articular cartilage of the femoral head. Several hunters reported that they first detected green discolouration in a small surface area that later expanded slowly. Cutting up the carcass revealed much more extensive discolouration and odour in the depth of muscles.

**Table 4:** Results of cultivation of bacteria from samples^a^ of spoiled moose meat

Sample No.	1	2		3		4		5		6	7	8		9	10	11	12	13
**Shot (date)**	08.10. 2008	12.10. 2008		02.10. 2009		12.10. 2009		07.09. 2010		06.09. 2011	06.10. 2011	11.10. 2011		28.09. 2015	2015	25.9. 2020	27.9. 2020	27.9. 2020
**County**	Fi	Fi		Fi		Tro		Fi		Fi	Fi	Fi		No	?	Tro	Tro	Tro
**ID in Additional file 1 (only from Finnmark)**	3	Not registered		16		-		22		29	34	35		-	-	-	-	-
**Meat spoilage detected (days after shooting)**	5	4		1		7		3		2	?	1		?	?	3	1	3
**Sample received (days after shooting)**	8	10		11		8		7		8	5	7		10	?	11	9	30
**Pre-analysis handling**	?	Frozen		Frozen		Delivered directly to lab		Mail with cooling		Mail without cooling	Mail without cooling	Express mail with cooling		Delivered directly to lab	Delivered directly to lab	Kept cool, delivered directly to lab		Frozen
**Sub-samples /sample sites**		Green^c^	Fresh^c^	Spoiled	Control^d^	Spoiled	Control^d^	Spoiled	Control^d^			Spoiled	Control^e^					
**Mixed aerobe bacteria**	+	+++	-	-	-	+++	-	+++	+	+++	(+)	+++	++	++	++	++	+++	++
***E. coli***	+					+++				+++		+++	++	++	++			
***Serratia *** **sp.**		+++																
***Pseudomonas *** **sp.**	+	+++								+++	N. e.			N. e.	N. e.	N. e.	N. e.	N. e.
**Gram positive cocci** ^**b**^		+++				+++		+++									+++	
***Clostridium perfringens***						+++			+	+++		+++						
**Swarming** ***Clostridium *** **sp.**		+++	++	+++		+++		+++		+++	+++	+++		+++^f^		+++	+++^g^	
**Indole reaction of ** ***Clostridium *** **sp. isolates**		Pos	Pos	Pos		Pos		Pos		Pos	Not tested	Pos and neg		Neg		Pos	Pos	

^a^ Samples received at the Norwegian Veterinary Institute, Tromsø. The samples originate from the three northernmost counties in Norway: Nordland (No), Troms (Tro) and Finnmark (Fi) in the years 2008–2020

(+), single colonies; +, sparse; ++, moderate; +++, rich or dominating;

N. e., Not examined (not cultured at 20 °C); Pos, positive; Neg, negative; ?, unknown

^b^ Gram positive cocci includes *Micrococcus* sp., *Staphylococcus* sp. and alfa-haemolytic *Streptococcus* sp

^c^ Two different sample sites, one with greenish discolouration, the other with fresh colour

^d^ Control samples taken from macroscopically unaffected parts of the carcass

^e^ Control sample from a different animal, shot the same day, treated in the same manner, not spoiled

^f^ Identified as *Clostridium septicum*

^g^ Identified as *Clostridium sordellii*

After skinning the carcass was hung up for tenderization, either as a whole carcass (26 cases) or divided into eight or nine major parts (19 cases). Hanging up a whole carcass was associated with later detection of meat spoilage (42%; 11/26 detected within 2 days), and higher frequency of total condemnation (80%; 20/25). Corresponding figures for hanging up the carcass in parts: 68% (13/19) detected within 2 days and 58% (11/19) total condemnation. The differences were not statistically significant (detection within 2 days P = 0.08, total condemnation P = 0.11).

Ambient temperature at shooting was reported by 38 hunters. It was below 5 °C in 11 cases (min − 2.5 °C), from 5 to 9 °C in 19 cases and 10 °C or above in 8 cases (max. 14 °C). Temperature at shooting did not show any clear influence on number of days until meat spoilage was detected (data in Additional file 1).

Temperature during the tenderization period was reported by 42 hunters. However, in 15 of these cases the meat spoilage was detected within 1 day, thus there was no tenderization period. In the remaining 27 cases the temperature was mainly below 5 °C in eight cases, 5–7 °C in ten cases and 8–10 °C in nine cases. There was a trend towards earlier detection of meat spoilage when temperature was higher: 5.3 days below 5 °C, 4.1 days at 5–7 °C, 3.9 days at 8–10 °C. The differences were not statistically significant (Additional file 1).

No hunting team had access to chilled facilities during the tenderization period, and the carcasses were exposed to changing temperatures between night and day, making temperature registrations less accurate.

Based on the reported temperatures during the tenderization period, an approximate number of day degrees could be estimated. In two cases the recommended 40 day degrees tenderization period was exceeded, and in one of these cases the description given in the report corresponds more to excess tenderization (meat slipping the bones), than the phenomenon of “green moose”.

The questionnaire did not ask for details about the killing of the moose, and 31 reports do not specify this. Three hunters reported that the moose had fallen after the first shot, but was not dead, and was euthanized with a second shot against the upper neck. One hunter reported that the moose fell immediately after shooting, while six hunters reported that the moose walked or ran for a distance, varying from 10 to 230 m, before falling dead. In two cases, the moose was injured, but not killed by the first shot. In one of these cases the moose was followed and killed by a shot to the upper neck 2–3 min later, still standing. In the other case the moose was tracked and shot dead two hours later. The injuring shots had not perforated the gastrointestinal tract in these two cases.

### Bacteriology

Samples of meat from 13 cases of meat spoilage was submitted to the NVI in Tromsø for analysis. The main results of bacteriological examination are presented in Table 4. Samples from each moose often consisted of 2–4 subsamples, including both spoiled and fresh muscles. Within samples from spoiled muscles there was variation between areas visibly discoloured and areas with a fresh colour, and sample sites sometimes included both kind of areas. The results in Table 4 try in part to summarize the findings from different subsamples and sample sites.

A mixture of aerobic bacteria was found in 12 of the 13 samples (92%). The amount of growth varied from single colonies (Sample No. 7) to rich growth. *E. coli* was identified in 6 samples while Gram positive cocci were dominating in 4 samples. *Pseudomonas* sp. was isolated in three cases, and isolates were identified as *Pseudomonas fluorescens* and *Pseudomonas putida*. Culturing at 20 °C was necessary to detect *Pseudomonas* sp., as none of the isolates were able to grow at 37 °C.

Swarming *Clostridium* sp. occurred in 10 of 13 samples (77%), and in two cases the swarming clostridia were the only (Sample No. 3) or nearly the only (Sample No. 7) bacteria growing. The swarming *Clostridium* sp. was most often indole positive, and was in one case (Sample No. 12) identified as *Clostridium sordellii*. Another attempt of identification (Sample No. 5) gave “unacceptable profile”. The isolate in Sample No. 5 was tested for its ability to grow at different temperatures, and was shown to grow well at 20 °C, but showed no growth at 4 °C. In Sample No. 10, the swarming clostridia were indole negative and identified as *Clostridium septicum*.

### Histology

**Fig. 3:**
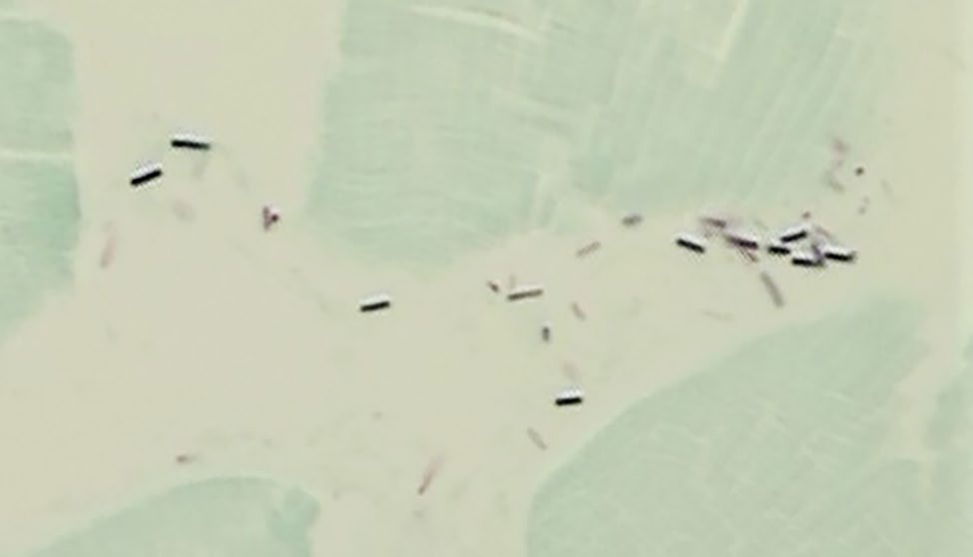
Gram positive rods in intermuscular connective tissue in a case of meat spoilage in an adult female moose. (Sample No. 2 in Table 4). Gram stain, counterstained with Fast green (muscle fibres stained green). Microphoto 630x

Samples of meat from seven of the cases examined by bacteriology (Samples No. 1–6 and 8 in Table 4) were also subjected to histological examination. The microscopical findings were similar in all cases. In samples from spoiled meat bacteria could be seen in ample amounts, most numerous in muscle fasciae and intermuscular connective tissue (Fig. 3). The histological appearance was otherwise normal, with no signs of inflammatory reactions, indicating that the bacterial invasion had happened post mortem.

## Discussion

The present report reviews the occurrence of sudden and unexpected meat spoilage during the moose hunting. The phenomenon occurs with low prevalence in northern Norway, estimated to 0.85% in Finnmark county. The moose show no signs of disease, neither when observed before shooting nor during evisceration and skinning. In more than 50% of cases the meat spoilage is detected within 2 days after shooting.

The estimated prevalence of 0.85% is uncertain. The more accurate registration of cases 2013–2021 resulted in exclusion of one case, and called a couple of other cases into question, as they may be caused by too long tenderization period. Thus, the total prevalence of 0.85% may be too high. On the other hand, unreported cases may also exist.

Based on the bacteriological and histological examinations, it seems fairly safe to conclude that the meat spoilage is caused by bacteria. But the bacteriological results are not easily interpreted in this report, for several reasons. First, the pre-analysis handling (sampling, storage and transport of samples) is highly variable (Table 4). Second, the SOP at NVI, Tromsø, is designed to detect pathogenic microbes, and is not ideal for analysis of food microbiology. And third, the selection of sample site is not standardized and both qualitative and quantitative results may vary according to sample site selection. Still, the results are included, as the information obtained is considered to be of some value, even with the weaknesses mentioned.

Contamination by bacteria commonly occurs on the surface of a carcass, and the aim of slaughter hygiene is to keep the number of bacteria as low as possible. Sauvala et al. [6] studied number of bacteria on the surface of moose carcasses in Finland, hunted and slaughtered under field conditions, and concluded that the hygienic standard was variable, but often poor. They state that the bacterial counts were so high that, according to EU legislation, 25% of the moose carcasses would be unacceptable due to high counts of mesophilic aerobic bacteria and 47% unacceptable due to high counts of bacteria in the family Enterobacteriaceae.

In the present report, a mixture of aerobic bacteria was detected in 12 of 13 samples. The sample site was from inside of the sample and not on the surface. Still, with often small muscle samples and variable handling of samples prior to analysis, the growth of aerobic bacteria is difficult to interpret, and may in part reflect surface contamination.

The finding of clostridia in 10 of 13 samples is more interesting, as clostridia are strict anaerobic and will not grow in the aerobic conditions on meat surface. Clostridia are spore-forming Gram positive rods, and some species are able to cause both greenish discolouration and putrid odour when growing in meat [8]. Their requirement of anaerobic conditions is consistent with the hunters’ observation that the greenish discolouration was found in depth of muscles where anaerobic condition may exist: close to the hip joint and femoral bone in the steak or close to the vertebral column in the neck and loin. Thus bacteria in the genus *Clostridium* may be suspected to be involved in this form of meat spoilage in moose.

The “green moose” cases are in some ways similar to the unique occurrence of meat spoilage in cattle in Belgium, caused by *Clostridium novyi* type B [9]. As for the cattle in Belgium, the “green moose” showed no signs of disease before shooting, and no signs of disease was discovered post-mortem. Further similarities are the development of spoilage in depth of muscles, the greenish discolouration and the foul odour.

But there are also differences. Eeckhaut et al. [9] detected only one species of clostridium, while in the “green moose” cases we have identified at least three species (*C. perfringens, C. septicum* and *C. sordellii*). Further, the spoilage in cattle occurred after several days of chilling at 7 °C, while more than half of “green moose” cases were discovered within two days after shooting, and five cases as early as within 5 h.

Eeckhaut et al. [9] found it unlikely that the meat spoilage in their cattle is caused by post-mortem contamination. They concluded that spores of *C. novyi* must have been present in the carcasses of the live animals, and, for unknown reasons, they germinated and grew in the carcasses post mortem.

The study of pathogenesis in black disease (necrotic hepatitis) in sheep (*C. novyi* type B) and blackleg in cattle (*C. chauvoei*) have revealed that clostridial spores may exist in a dormant state for long time (months) in macrophages in liver and muscle, and possibly other tissues [10, 11]. Experiments on mice have shown that intravenously injected spores of *C. perfringens* may settle in liver and are eliminated with a half-life of 6 days [12]. In vitro experiments have shown that both vegetative cells and spores of *C. chauvoei* remain viable in murine and bovine macrophages [13]. The vegetative cells in murine macrophages, but not bovine, showed significant decline in number during the experimental period (72 h), while spores did not.

The occurrence of dormant clostridial spores in moose has not been studied, but it is not unlikely that such spores may exist. A common source of dormant spores, like Eeckhaut et al. [9] suggest for the cattle in Belgium, may explain the sudden occurrence of multiple cases in the same area same year. However, the possibility that “green moose” is caused by sudden germination of dormant spores is contradicted by the extremely rapid development of the meat spoilage in many of the cases. The revival time of spores of *Bacillus subtilis* is reported to be 2.5 h [14], and if the revival time is similar in clostridia, it seems unlikely that germinating spores should be able to cause odour and greenish discolouration within 5 h from shooting, as have been reported by the hunters in five cases of “green moose”.

Another possible explanation may be that vegetative clostridia are present in the muscle prior to shooting. Deep tissues of healthy animals are normally considered to be sterile, though it is generally accepted that a small number of clostridia may occur in some animals, mainly in liver, spleen and lymph nodes, disputably also in muscle [15, 16]. As with germination of spores it seems unlikely that a very small number of intrinsic bacteria should be able to cause such rapid meat spoilage as reported by the hunters.

Dissemination of gastrointestinal bacteria in the circulation in close connection to the shooting would better explain the meat spoilage as observed. Very rapid meat spoilage could then be explained by massive spread of bacteria during the killing, while less severe cases could be caused by smaller amounts of bacteria, reaching only some parts of the carcass. From a theoretical point of view, spread of gastrointestinal bacteria by blood might happen through lesions or changes of permeability in the gastrointestinal tract [16], but no reports confirms that this actually happens in practice. It is also difficult to see a reason for sudden occurrence of several cases of “green moose” in the same area same year if meat spoilage is caused by random spread of gastrointestinal bacteria during killing.

Different kinds of possibly predisposing events occurred during shooting, evisceration and skinning of the moose that became spoiled, e.g., hit point outside preferred target area, injured but not killed by first shot, evisceration later than 60 min after shooting, contamination by ruminal content and delayed skinning. For most of these events reference data are lacking, making it difficult to interpret the significance of the events. E. g. delayed skinning occurred in 66% (29/44) of meat spoilage cases. Keeping the fur on the carcass for prolonged time will delay cooling, and temperature is an important factor for bacterial growth. But the significance of delayed skinning to this form of meat spoilage is uncertain, as we have no data to sustain that “green moose” occur relatively more often after delayed skinning than in animals skinned immediately after shooting. The hunters claim that postponing the skinning until next day is common practice, both to increase tenderness and avoid cold shortening during cold weather [17], and to protect the carcass during transport. Besides, 15 cases of meat spoilage are reported in carcasses skinned within few hours after shooting, showing that delayed skinning is not a prerequisite for this type of meat spoilage.

When it comes to injury shootings two of 45 “green moose” (4.4%) were injured after first shooting (3 of 46; 6.5% if the excluded case is taken into account). The Norwegian Association of Hunters and Anglers made a questionnaire study of moose hunting [18]. In this study injury shootings were reported to have happened in 5.9% (443/7546) of moose shootings. In a Swedish questionnaire study, 10.3% (180/1746) of moose shootings resulted in suspected injury shooting, and tracing of the moose by dog [19]. Based on these results there are no reasons to believe that injury shootings are more frequent in “green moose” than during moose hunting in general.

The questionnaire did not ask for details about the killing of the moose (number of shots, behaviour after the shot). In view of the possibility that bacteria are spread during killing, questions concerning the killing and bleeding should have been included. Still, the lack of reference data would have made interpretation difficult.

A significant finding in this report is that adult bulls are more frequently and calves less frequently subjected to “green moose” meat spoilage. Several factors may be discussed as possible causes of this finding. Adult bulls are heavier and calves lighter than the other sex/age classes. This may influence the possibility of maintaining slaughter hygiene as suggested by Sauvala et al. [6]. Large and more muscular carcasses are also more slowly cooled than smaller carcasses, and any existing fat layer will utterly slow the cooling process [17]. This favours bacterial growth in bulls and not in calves. The “green moose” meat spoilage predominantly arise in deep muscles (neck, thigh); areas that will also retain body temperature for longer time after shooting. Thus, the slower cooling of deep muscles in more muscular individuals may possibly explain the differences in occurrence of “green moose” meat spoilage between sex and age classes.

Hunting season is also rutting season in moose, and in adult bulls the rut is accompanied by reduced feed intake [20], increased levels of testosterone [21] and possibly also increase in cortisol, as has been documented in male reindeer during rut [22]. It may be speculated if these behavioural and hormonal changes may predispose to the bacterial meat spoilage in adult bulls, but it is difficult to see how these factors explain the clearly lower prevalence in calves.

Temperature is a critical factor for bacterial growth, and questions about ambient temperature were included in the questionnaire to explore the possible influence of ambient temperature on the occurrence of “green moose” meat spoilage. However, our data do not support that ambient temperature is important for the occurrence of “green moose”. The temperatures reported seem to represent normal variation of autumn temperatures in Finnmark county. In addition, some cases arise so rapidly that the ambient temperature is unlikely to have major influence compared to internal carcass temperature. For cases detected after 2 days or more, we used number of days until detection of meat spoilage as a measure of temperature influence, without finding significant differences. The trend of earlier detection at higher temperatures may be due to the earlier cutting of the carcass (shorter tenderization period) at higher temperatures, as many of the cases detected after several days were detected during cutting of the carcass.

The average temperature (1991–2020) in major hunting municipalities like Deanu/Tana and Kárásjoga/Karasjok is 3 to 3.5 °C higher in second half of September, compared to first half of October [23]. Still, the monthly distribution of “green moose” cases in Finnmark closely resembled the shooting of moose in general, supporting the impression that “green moose” is not primarily a result of high ambient temperature.

## Conclusions

This report describes a particular form of bacterial meat spoilage occurring in moose shortly after shooting. Bacteriological examination of submitted samples was inconclusive, but showed the presence of different gastrointestinal bacteria, including clostridia. Based on the characteristic features of this meat spoilage - greenish discolouration and foul odour arising from the depth of muscles - we suggest that clostridia are a main factor involved. But how and why clostridia are spread to the muscles and causing such rapid meat spoilage, remain unsolved.

## Electronic supplementary material

Below is the link to the electronic supplementary material.

**Additional file 1.** Three tabs named Carcass data, Carcass weights and Ambient temperature

## Data Availability

The dataset used in the current report are available from the corresponding author on reasonable request.

## Declarations

### Prior publication

Data have not previously been published.
